# Mode I Crack Propagation Experimental Analysis of Adhesive Bonded Joints Comprising Glass Fibre Composite Material under Impact and Constant Amplitude Fatigue Loading

**DOI:** 10.3390/ma14164380

**Published:** 2021-08-05

**Authors:** Alirio Andres Bautista Villamil, Juan Pablo Casas-Rodriguez, Alicia Porras Holguin, Maribel Silva Barrera

**Affiliations:** 1 Structural Integrity Research Group, Department of Mechanical Engineering, Universidad de los Andes, Bogotá 111711, Colombia; aa.bautista@uniandes.edu.co; 2 Grupo de Diseño de Productos y Procesos (GDPP), Department of Chemical and Food Engineering, Universidad de los Andes, Bogotá 111711, Colombia; n-porras@uniandes.edu.co; 3 CIPAER Group, Escuela de Postgrados Fuerza Aérea Colombiana EPFAC, Bogotá DC 110111, Colombia; maribel.silva@fac.mil.co

**Keywords:** adhesive-bonded joints, crack propagation, composite materials, double cantilever beam, impact fatigue, constant amplitude fatigue

## Abstract

The T-90 Calima is a low-wing monoplane aircraft. Its structure is mainly composed of different components of composite materials, which are mainly bonded by using adhesive joints of different thicknesses. The T-90 Calima is a trainer aircraft; thus, adverse operating conditions such as hard landings, which cause impact loads, may affect the structural integrity of aircrafts. As a result, in this study, the mode I crack propagation rate of a typical adhesive joint of the aircraft is estimated under impact and constant amplitude fatigue loading. To this end, effects of adhesive thickness on the mechanical performance of the joint under quasistatic loading conditions, impact and constant amplitude fatigue in double cantilever beam (DCB) specimens are experimentally investigated. Cyclic impact is induced using a drop-weight impact testing machine to obtain the crack propagation rate (da/dN) as a function of the maximum strain energy release rate (G_Imax_) diagram; likewise, this diagram is also obtained under constant amplitude fatigue, and both diagrams are compared to determine the effect of each type of loading on the structural integrity of the joint. Results reveal that the crack propagation rate under impact fatigue is three orders of magnitude greater than that under constant amplitude fatigue.

## 1. Introduction

Composite materials are widely used in aircraft structures mainly due to their exceptional stiffness-to-weight ratio. This characteristic makes them more attractive than other materials for applications in which low-weight requirements and high-stiffness conditions are desirable, predominantly in cases where fuel or energy consumption is directly related to the structure weight [1].

Since the development of the aircraft industry, various methods have been employed to join aircraft components; however, the broad use of composites in the last two decades has addressed interests of engineers and researchers for employing adhesives as a potential joining technology [2].

The adhesive bonding process permits the joining of substrates with an adhesive between them. Compared to the conventional mechanical fastener method, this method provides several advantages, including lower structural weight, reduced stress concentrators and higher performance under fatigue conditions. All of these factors in turn lead to the increase in the structural integrity of the system [3]. Nevertheless, the adhesive bonding process exhibits some disadvantages. The reliability of the method considerably depends on the quality of the prepared surface. Moreover, adhesives are susceptible to environmental degradation and are perishable materials; thus, the mechanical strength of joints can be affected by the foregoing factors. Finally, defects in adhesives can be evaluated by nondestructive testing, but methods are not reliable for determining strength; hence, destructive tests should be employed instead of nondestructive ones [4].

Trainer aircrafts may be exposed to adverse operating situations, such as hard landings, which are characterized by a ground approach with a vertical speed greater than 3 m/s [5]. During landing, the main landing gear first impacts the ground, and the force generated by the impact is transferred to the wings, as the main landing gear in low-wing monoplane aircrafts is supported by the wing beam. Hence, the entire structure absorbs the energy induced during landing [6].

The aircraft is mainly manufactured using composite materials assembled by adhesive joints. The adhesive comprises a mixture of an epoxy resin or a hardener and a filler substance, which is a flocked cotton fibre. This filler makes the mixture consistent, thereby easing the application of the adhesive to the structure. The adhesive thicknesses of the main aircraft joints are 3.18 mm to 12.70 mm; hence, it is important to analyse the dynamic response of adhesive joints with respect to changes in thickness, thus the adhesive thicknesses of DCB specimens used in this study are in the mentioned interval due to a requirement of the actual manufacturing process of the aircraft. Moreover, the most critical joint of the aircraft is located in the support joint between the fuselage and wing beam; thus, it is crucial to obtain knowledge about the mechanical behaviour of a typical adhesive joint of an aircraft when a crack appears and starts to propagate inside it [7].

Fracture toughness of an adhesive joint depends on the bond line thickness during the propagation of the crack inside the adhesive [8]. This can be explained on the basis of the development of a plastic zone at the crack tip. When the bond line thickness is less than the diameter of the plastic zone, the development of the plastic zone at the crack tip is restricted, resulting in low fracture toughness [9]. With the increase in the bond line thickness, substrates exert a low constraint on the development of the plastic zone; hence, fracture toughness increases until the complete development of the plastic zone at the crack tip [10]. At this point, the distortion of the stress distribution at the crack tip can occur, modifying the shape of the plastic area and converting it into an ellipse; thus, the fracture toughness increases due to the distribution of the stress in a large area. At a considerably high bond line thickness, the diameter of the plastic zone at the crack tip is completely developed due to the decrease in the constraint as the presence of stiff adherends is not sufficient to restrict the growth of the plastic zone, thereby reducing the length of the plastic zone and resulting in low fracture toughness [11]. The crack growth rate da/dN, where a is the crack length, and N is the number of cycles, depends on the amount of plastic deformation; hence, in general, a higher adhesive thickness leads to lower constraint and subsequently a larger plastic deformation; hence, crack growth rates are lower. Additionally, a higher crack growth rate resistance is correlated with higher fracture toughness [12].

Impact fatigue is induced by low-energy, low-velocity impacts, which detrimentally affect the performance and reliability of components [13], and may cause internal damage in the form of delamination, crack formation and fibre breakage, rendering few visual signs on the external surface of the damaged material [14]. Notably, few studies investigated the effects of impact fatigue phenomenon on the structural integrity of adhesive joints [15]. However, important research regarding the understanding of the crack behaviour of adhesives and adhesive joint systems in composite materials has been reported.

Carbon-fibre-reinforced polymers (CFRPs) were tested under a mixed mode impact and constant amplitude fatigue loading, and results revealed that the crack propagation rate is highly dependent on the fracture mode; under repeated impact, the rate is greater than that under constant amplitude fatigue [16]. A crack growth law for impact fatigue was developed [17], on the basis of the relation between the maximum dynamic strain energy release rate and the impact fatigue crack growth rate in an epoxy adhesive FM73. Another study analysed the delamination failure mechanism in bonded composite joints, and results revealed that propagation occurs as a result of the interaction of several fracture mechanisms, which are significantly affected by the type of the applied load; in summary, impact fatigue causes more damage than constant amplitude fatigue [18]. Similarly, the response of lap strap joints comprising CFRP bonded with a rubber-modified epoxy adhesive under constant amplitude sinusoidal loading, cyclic in-plane impact and their combination was investigated. Two main failure patterns were noted: Initially, cohesive failure in the adhesive with low fatigue crack propagation rates and then failure of the adhesive and CFRP, with an acceleration of the crack growth during the transition of one failure pattern to the other one under the constant amplitude fatigue regime. Introduction of a small number of impacts between constant amplitude sinusoidal loading blocks led to the increase in the crack propagation rate [19].

Furthermore, DCB specimens were tested under impact fatigue and constant amplitude fatigue loading. The crack propagation rate was one order of magnitude lower under constant amplitude fatigue loading for the same range of the maximum strain energy release rate; hence, it is imperative to examine this type of loading for the structural integrity analysis of an adhesive joint [20,21]. The fracture toughness also depends on the type of adhesives and substrates of the bonded system. The fracture toughness of brittle adhesives exhibit low dependence on the strain rate; nevertheless, the fracture toughness of ductile adhesives is more likely affected by the loading condition than that of brittle ones [22]. Impact strength of a joint comprising composite substrates is highly dependent on substrate properties [23]. Previously [24], joints manufactured using carbon-fibre substrates with different fibre orientations were subjected to impact load, and results revealed that irrespective of differences between stacking sequences of substrates, joint failure occurs due to the delamination of the substrate. In addition, the rate dependency of the structural integrity of the joint was mostly caused by the composite resin matrix [25,26]. Impact tests of adhesive bonded joints also have been performed using dissimilar substrates. In [27], the dynamic strength of single lap joints with steel and aluminium substrates was evaluated, and results revealed that the less stiff substrate determines the strength of the joint and energy absorption; thus, properties of substrates play a key role in the structural integrity of adhesive joints [28].

Drop-weight impact test (DWIT) is one of the methods that is employed for impact tests. DWIT can characterise materials under a low-velocity impact of ≤10 m/s and strain rates of up to 102 s^−1^ [29]. In this study, DWIT was employed to propagate cracks in DCB specimens under mode I loading to obtain diagrams of the crack propagation rate (da/dN) as a function of the maximum strain energy release rate (G_Imax_).

In this paper, the effect of the adhesive bond thickness on the mechanical performance of DCB specimens under mode I quasistatic, cyclic impact and constant amplitude sinusoidal loading conditions was analysed. For this purpose, the fracture toughness and crack growth rate under the mentioned dynamic loading conditions were measured by using an adhesive and adherends of a typical joint system of the T-90 Calima aircraft. Details of the materials and geometry of the specimens as wells as characterization techniques are given in the materials and methods section, in addition, crack growth and fracture toughness diagrams are presented in the results section and are divided according to the type of loading the specimens were subjected to, and, to conclude, the effect of the adhesive bond thickness on the crack propagation rate and strain energy release rate is discussed.

## 2. Materials and Methods

The adherends of the specimens comprised a glass-fibre cloth as the dispersed phase and an epoxy laminating resin as the continuous phase or matrix. The laminates were stacked using a wet lay-up procedure with eight plies and a ±45° sequence and cured at 66 °C for 15 h. The nominal thickness of each fibre reinforced polymer (FRP) panel was 3 mm. The adhesive comprised an epoxy resin and a filler substance called a flocked cotton fibre, which increased its viscosity and enabled it to be applied to surfaces with specific thicknesses [7].

**Figure 1:**
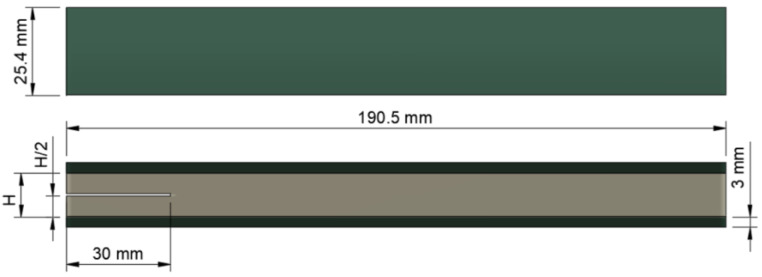
Schematic representation of a DCB specimen.

Dimensions of DCB specimens as well as the test method were based on the ASTM D5528 [30], which describes the determination of the opening mode I interlaminar fracture toughness G_IC_. Dimensions of the DCB specimens (Figure 1) included a width of 25.4 mm, a length of 190.5 mm with a precrack of 30 mm located in the middle of the adhesive layer, which started at one end of the specimen. Precracks were generated by means of 0.5 mm polymer sheets and located within the adhesive using aluminium spacers according to the nominal thickness of the joint (H). DCB specimens were manufactured using three thicknesses for the adhesive bonded joints: 3.18 mm, 6.35 mm and 12.70 mm and aluminium spacers were also used in this step to separate FRP panels and give the desire adhesive joint thickness to DCB specimens. A curing time of seven days at 20 °C was used before the specimens were removed from their mould and were cut to final dimensions by means of high-pressure water jet machine.

The opening mode I interlaminar fracture toughness was determined by the modified beam theory (MBT) method (Equation (1)). Large displacement effects were corrected by the inclusion of a parameter F (Equation (2)) in the calculation of G_IC_. The specimen was loaded at a constant crosshead rate of 1 mm/min in an Instron 3367 universal testing machine. Crack length was measured by two methods: visual measurement with graduated marks along each of the specimens and throughout the specimens using Kyowa crack propagation gauges.

(1) $$G_{IC}=\frac{3P\delta }{2b\left(a+\Delta \right)}F$$

(2) $$F=1-\frac{3}{10}{\left(\frac{\delta }{a}\right)}^{2}-\frac{3}{2}\frac{\delta t}{a^{2}}$$

where *P*-load, δ-load point displacement, *b*-specimen width, *a*-crack length and *t*-distance between the attachment point of the piano hinge and a quarter of the total specimen thickness including the adhesive and substrates.

Δ was experimentally determined by generating a least-squares plot of the cube root of compliance C^1/3^ as a function of crack length; thus, Δ is the distance between the origin and the intercept of the linear interpolation of C^1/3^ with the abscissa. The compliance C is the ratio of the load point displacement to the applied load δ/*P*.

Cyclic impact tests were performed using a drop-weight impact testing machine. Details of tests are described in [7]. DCB specimens used for cyclic impact tests had to be stiffened by adhering AISI 1020 steel plates with a thickness of 4.76 mm on the upper and lower surfaces of the specimen to reduce the load point displacement per cycle. A diagram of the crack propagation rate (da/dN) as a function of the mode I maximum strain energy release rate (G_Imax_) was obtained by plotting the derivative of the crack length (*a*) as a function of the number of impacts. G_Imax_ was obtained by the application of the previously explained MBT method. The crack length was measured by visual inspection with graduated marks along the specimen length.

**Figure 2:**
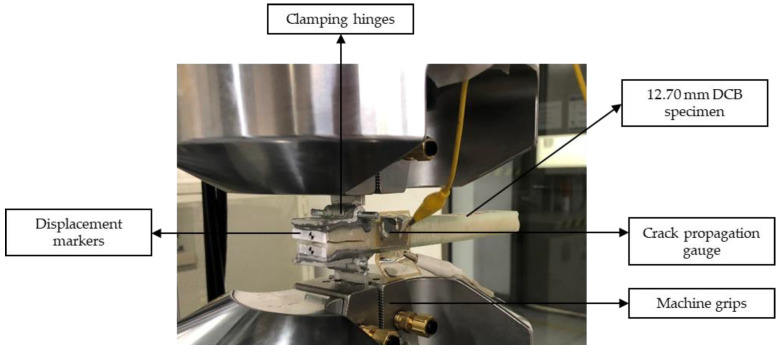
Experimental setup for crack propagation tests under constant amplitude fatigue.

Crack propagation tests under constant amplitude fatigue were performed in a servo hydraulic MTS 370 axial fatigue machine under the displacement control mode, inducing a constant amplitude sinusoidal waveform using a peak-valley compensator. Figure 2 shows the experimental setup. The displacement ratio was 0.1, and the load point displacement was measured by adhering markers to the specimens and monitoring them using a high-resolution video camera. Crack propagation gauges were stuck on one side of the DCB specimens to measure the crack length per cycle. The opening load was applied by adhering piano hinges to both surfaces of the specimen using an epoxy adhesive.

The fracture surface was observed by scanning electron microscopy (SEM) equipped with back-scattering electron imaging. SEM images were recorded on a JEOL scanning electron microscope (model JSM 6490-LV). Five images were recorded per specimen in different zones of the failure surface at 300× magnification, a voltage of 20 kV and a vacuum pressure of 20 Pa. The failure mechanism was classified according to the ASTM 5573 constant amplitude [31].

## 3. Results

### 3.1. Mode I Critical Strain Energy Release Rate Determination

**Figure 3:**
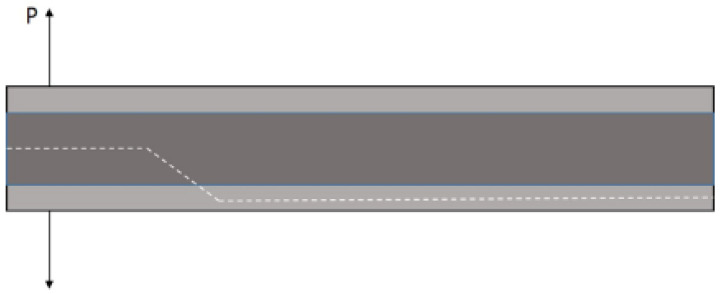
Schematic of the crack growth path in DCB specimens.

Opening quasistatic load was applied to 3.18-mm-, 6.35-mm- and 12.70-mm-thick DCB specimens to obtain the mode I critical strain energy release rate for each specimen. Five repetitions per adhesive thickness were performed. In all of the examined specimens, a crack starts to propagate inside the adhesive due to induced precracks; nevertheless, after some crack increase, it changes its direction of propagation until the crack reaches one of the substrates of the specimens, delaminating it and growing between the substrate plies until failure (Figure 3).

**Figure 4:**
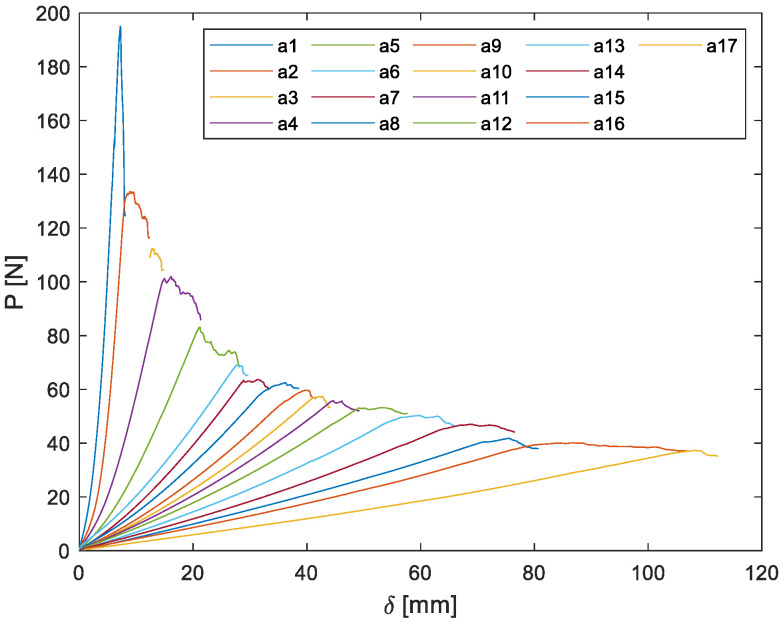
Characteristic diagram of a typical load displacement curve of a 6.35-mm-thick DCB specimen.

Figure 4 shows the force vs. load point displacement plot for a 6.35-mm-thick DCB specimen, which was obtained by loading and unloading the same specimen many times until complete debonding of one of the adherends from the system. Each colour of the curve represents different loading cycles. When a load drop off occurred, cracks propagated along the composite and the specimen was unloaded totally after measuring the crack length, then a new loading cycle started. A change in the slope of each loading cycle is observed owing to a stiffness degradation of the specimen with crack evolution, thus a lower peak force is required to propagate the crack as it grows.

**Figure 5:**
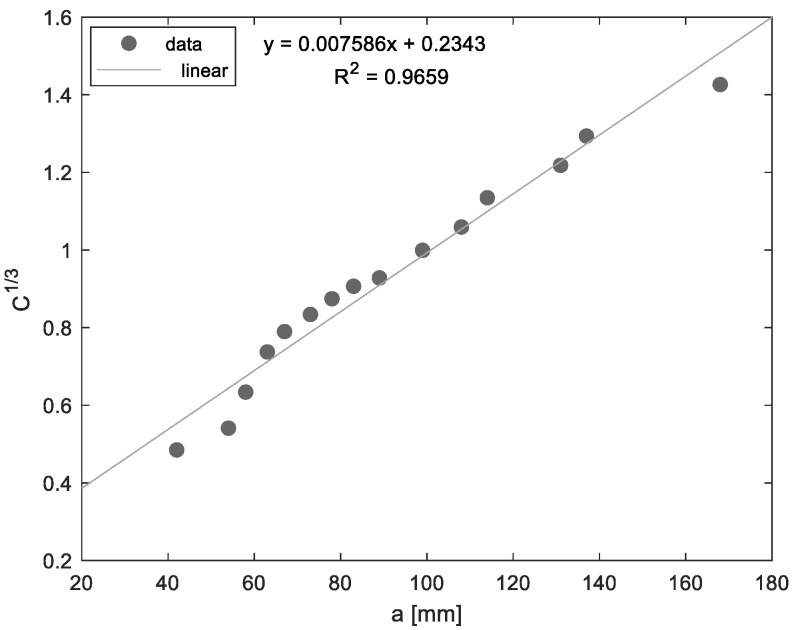
Linear interpolation of the cubic root of compliance as a function of the crack length.

Figure 5 shows the typical linear fitting curve of the cubic root of compliance as a function of the load point displacement. The intercept of the linear interpolation of C^1/3^ with the abscissa affords Δ, which is used as a correction factor for the mode I critical strain energy release rate due to the rotation that occurs at the delamination front of the specimen.

**Figure 6:**
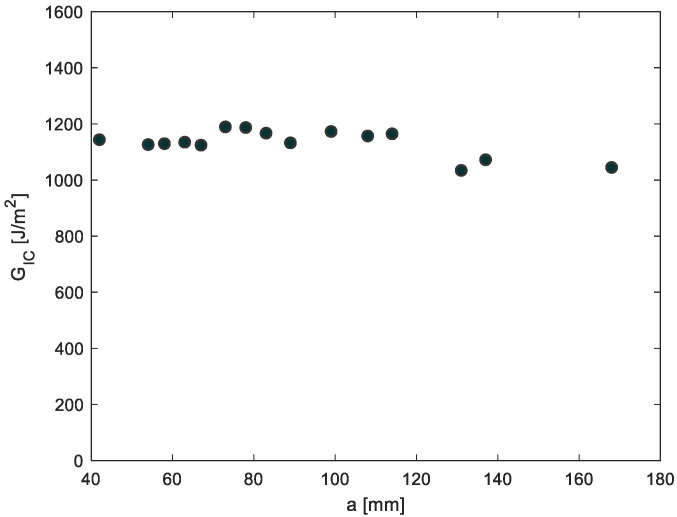
Mode I critical strain energy release rate as a function of the crack length for a 6.35-mm-thick DCB specimen.

**Figure 7:**
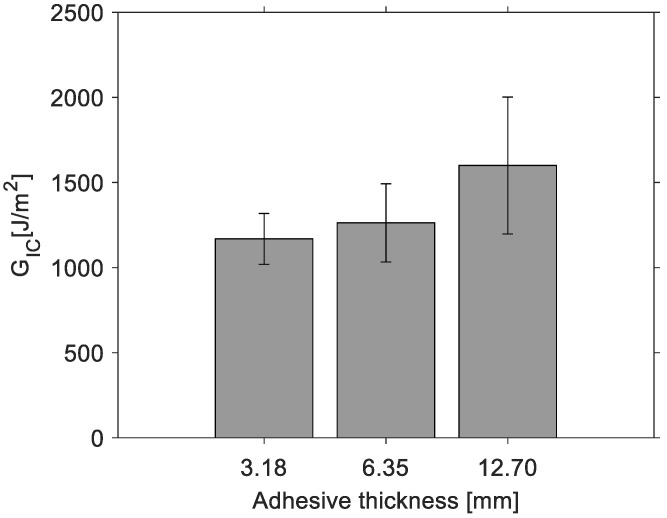
Mode I critical strain energy release rate.

Figure 6 shows G_IC_ values for each loading cycle shown in Figure 4 for a DCB specimen with an adhesive thickness of 3.18 mm in terms of the crack length. The final fracture toughness G_IC_ for each specimen is calculated from the average G_IC_ values for each loading cycle. From Figure 7, the average fracture toughness G_IC_ increases with the increase in the adhesive thickness; nevertheless, due to scatter, a significant difference between means is not observed.

### 3.2. Crack Propagation Rate Test under Dynamic Loading Conditions

Mode I crack propagation rate (da/dN) as a function of the maximum strain energy release rate G_Imax_ diagrams were obtained under repeated impact and constant amplitude fatigue to compare the influence of the load in the crack propagation behaviour. G_Imax_ was computed with the peak force of each cycle for both types of dynamic loads. Nine specimens were tested for each loading type, three specimens per nominal adhesive thickness. The crack growth path for all the specimens is similar to that shown in Figure 3, explained by the fact that the mode I fracture toughness of the bulk adhesive was considerably greater than that of the glass-fibre composite laminate; hence, it is the easiest route for crack growth.

**Figure 8:**
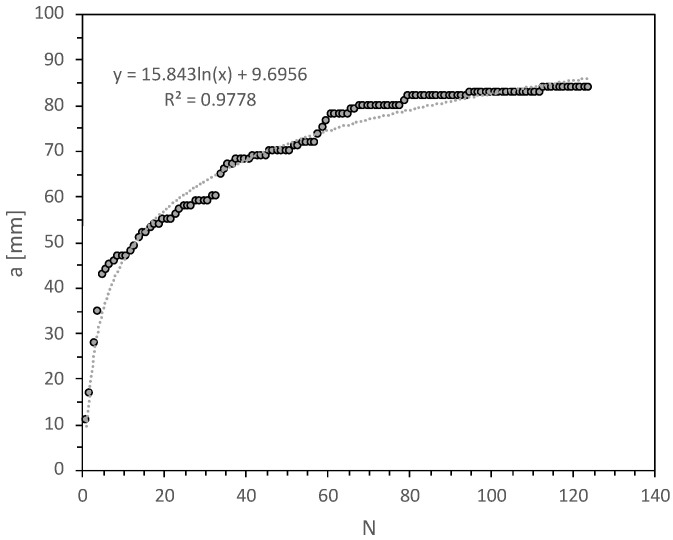
Crack length as a function of the number of impacts—impact fatigue.

**Figure 9:**
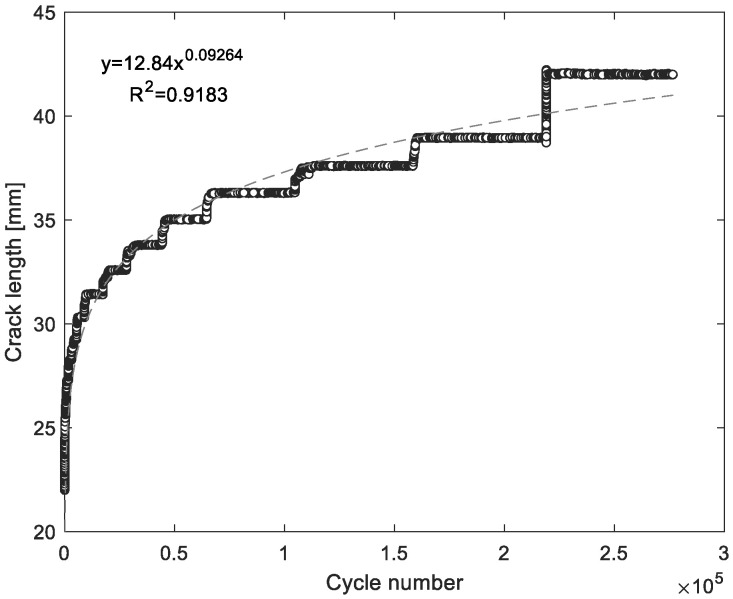
Crack length as a function of the number of cycles—constant amplitude fatigue.

Figure 8 and Figure 9 show the typical curves of the crack length (*a*) vs. the number of impact (N) or vs. number of cycles in the case of constant amplitude fatigue, showing logarithmic and power growth under repeated impact and constant amplitude fatigue respectively. This suggest that the mode I crack propagation rate (da/dN) is high at the first cycles and starts to decrease in a nonlinear manner until specimen failure, considering that da/dN was obtained by deriving the function found for the crack length vs. number of cycles plots. This behaviour was expected for both types of dynamic loads. Regarding constant amplitude fatigue, it is evident that the displacement control mode produces initially in the specimen a large crack length, which is proportional to the applied constant load point displacement, but then this constant opening will lead to a reduction in the crack growth during each cycle. Moreover, is important to notice that the real measurement of the change in the crack length corresponds to each step shown in Figure 9. Thus, the instantaneous crack length between steps is unknown due to the crack gauge. As a crack progresses, the grid lines of the crack gauge, which are separated one after another, are being broken, and an output value changes in the process, giving a reading of the crack evolution only when each grid is damage. Remarkably, an increase in the load point displacement generates a major deflection on substrates, absorbing an important fraction of the energy given by the impact phenomenon, which results in low crack propagation rates.

**Figure 10:**
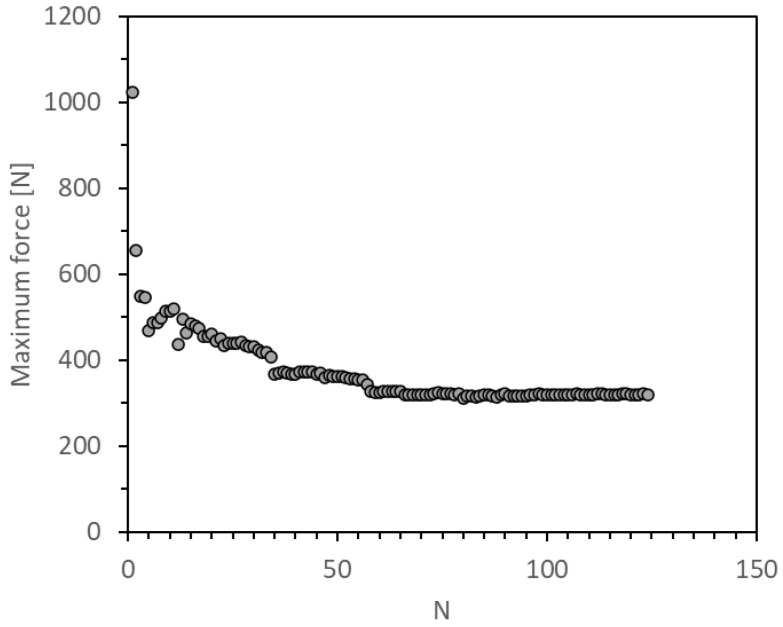
Evolution of the maximum impact force.

**Figure 11:**
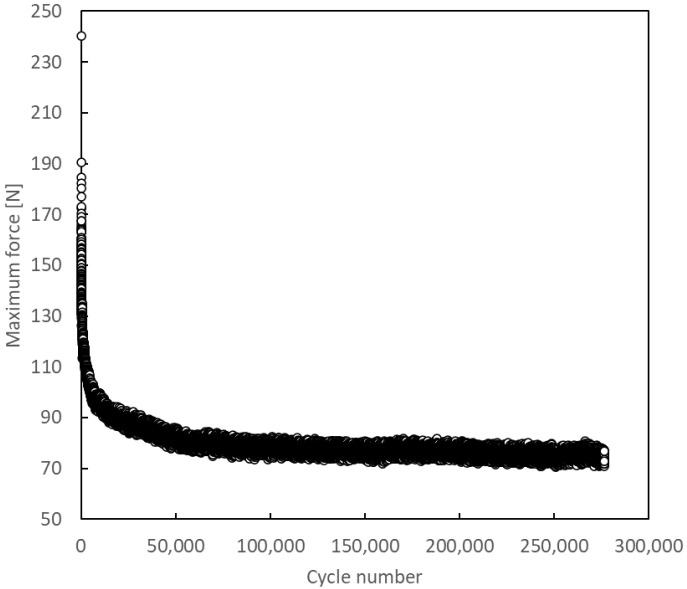
Evolution of the maximum force per cycle.

Figure 10 and Figure 11 show the evolution of the maximum dynamic load from which G_Imax_ was determined. It can be observed a sudden load-drop in the first cycles for both plots, followed by a levelling to a constant value. This behaviour could be related with the fact that the crack is initially in the middle zone of the adhesive, thus the force required to propagate a crack when its tip remains in the bulk adhesive is much higher than the required once the crack is between the substrate’s plies.

**Figure 12:**
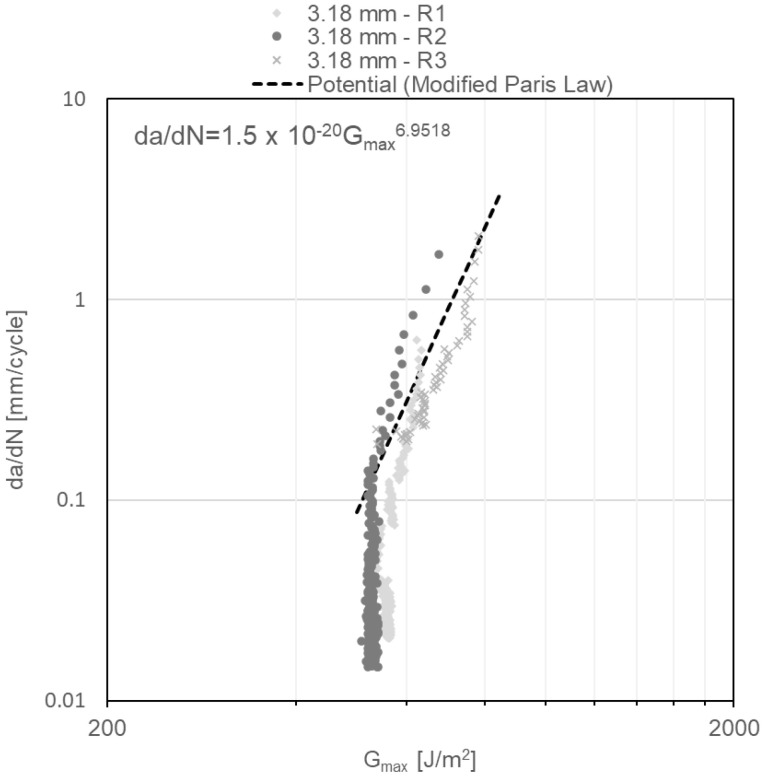
G_Imax_ vs. da/dN diagram for 3.18-mm-thick DCB specimens under impact fatigue.

**Figure 13:**
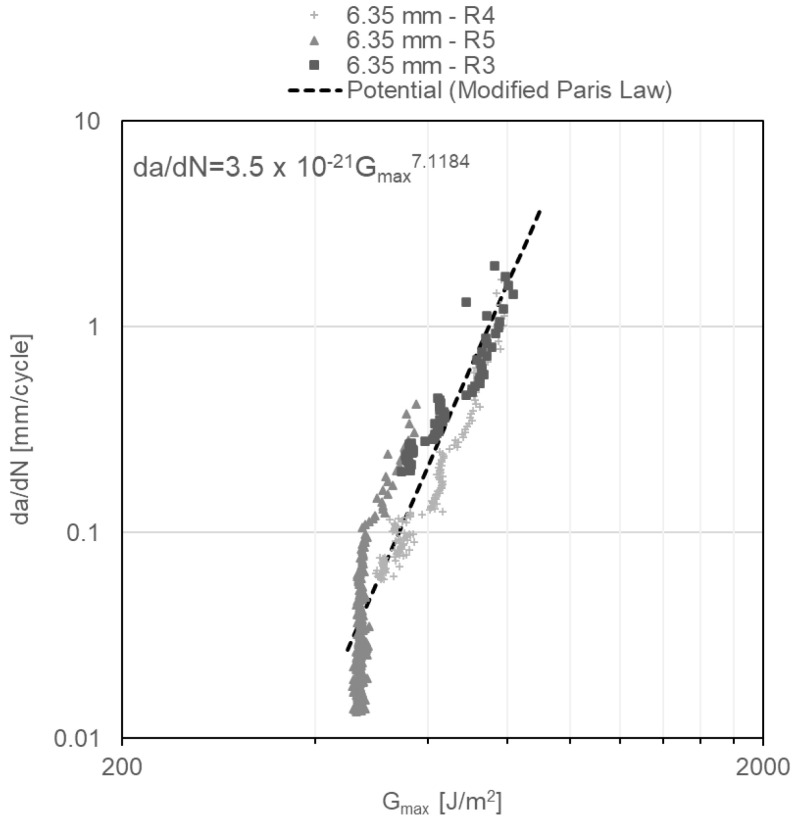
G_Imax_ vs. da/dN diagram for 6.35-mm-thick DCB specimens under impact fatigue.

**Figure 14:**
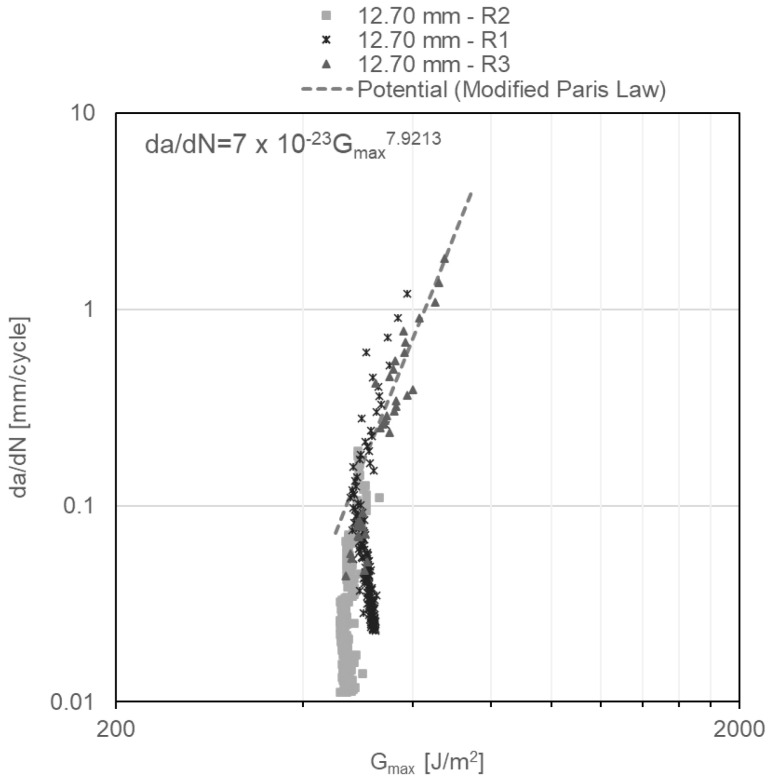
G_Imax_ vs. da/dN diagram for 12.70-mm-thick DCB specimens under impact fatigue.

Finally, da/dN as a function of G_Imax_ diagrams obtained under repeated impact, and shown in Figure 12, Figure 13 and Figure 14, reveal two regions of the crack behaviour. Region I is located beneath thresholds of 541 ± 17.8 J/m^2^, 471 ± 4.9 J/m^2^ and 490 ± 20.5 J/m^2^ for adhesive thicknesses of 3.18 mm, 6.35 mm and 12.70 mm, respectively, below which the crack does not grow. In region II, stable crack propagation is observed, where da/dN follows a power law shown in Equation (3), widely known as the modified Paris law:

(3) dadN=C·GImaxm

where C and *m* are material constants. For each of examined DCB specimens, the power law that describes the crack propagation behaviour is shown in the upper left corner of Figure 12, Figure 13 and Figure 14, which are obtained from the power-law fitting of the points in Region II of those figures. Notably, with the increase in the specimen thickness, the *m* constant in the modified Paris law equation increases, showing a slightly more susceptibility to increase the propagation rate with minor changes in G_Imax_ when the adhesive layer is thicker.

**Figure 15:**
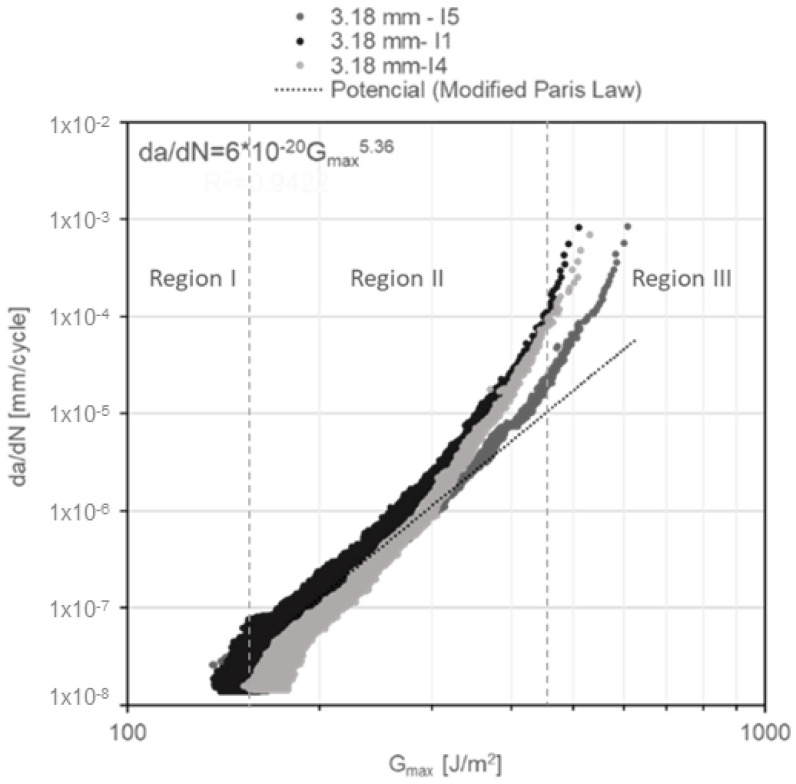
G_Imax_ vs. da/dN diagram for 3.18-mm-thick DCB specimens under constant amplitude fatigue.

**Figure 16:**
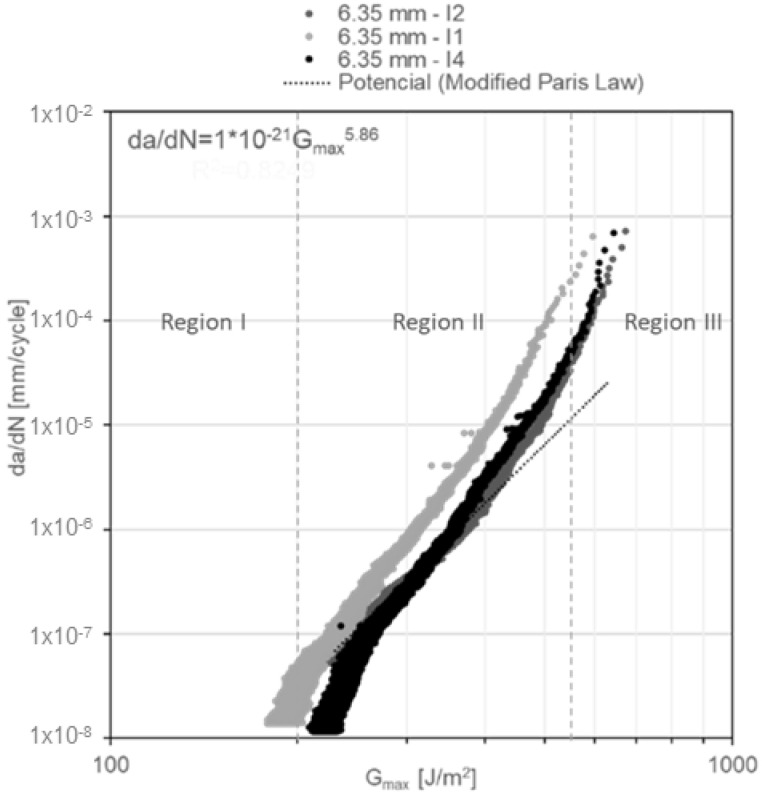
G_Imax_ vs. da/dN diagram for 6.35-mm-thick DCB specimens under constant amplitude fatigue.

**Figure 17:**
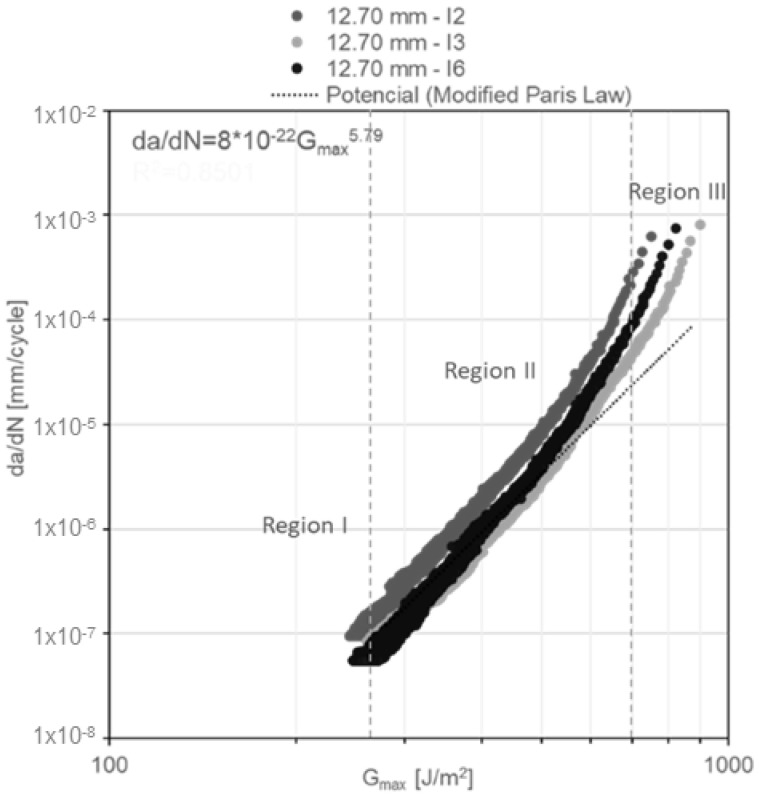
G_Imax_ vs. da/dN diagram for the 12.70-mm-thick DCB specimens under constant amplitude fatigue.

In addition, the da/dN diagrams as a function of G_Imax_ for constant amplitude fatigue (Figure 15, Figure 16 and Figure 17) reveal the failure mechanism of three regions. Region I is located beneath a threshold of 156 ± 11.7 J/m^2^ for DCB specimens with a nominal adhesive thickness of 3.18 mm; a threshold of 200 ± 19.4 J/m^2^ for DCB specimens with a nominal adhesive thickness of 6.35 mm and a threshold of 255 ± 3.7 J/m^2^ for DCB specimens with a nominal adhesive thickness of 12.70 mm. Region II exhibits stable crack propagation, where da/dN follows the modified Paris law as mentioned previously. Region III, where crack propagation starts to become unstable, is noted in the first cycles of Figure 15, Figure 16 and Figure 17. This change of slope in the diagram starts to show at ~40% underneath the critical energy release rate value.

**Figure 18:**
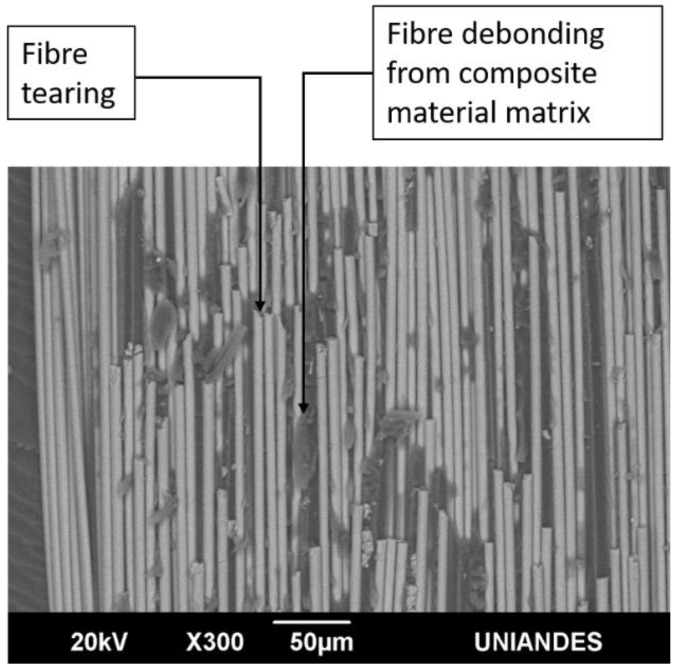
SEM fracture surface image of the 6.35-mm-thick I4 DCB specimen tested under constant amplitude fatigue.

**Figure 19:**
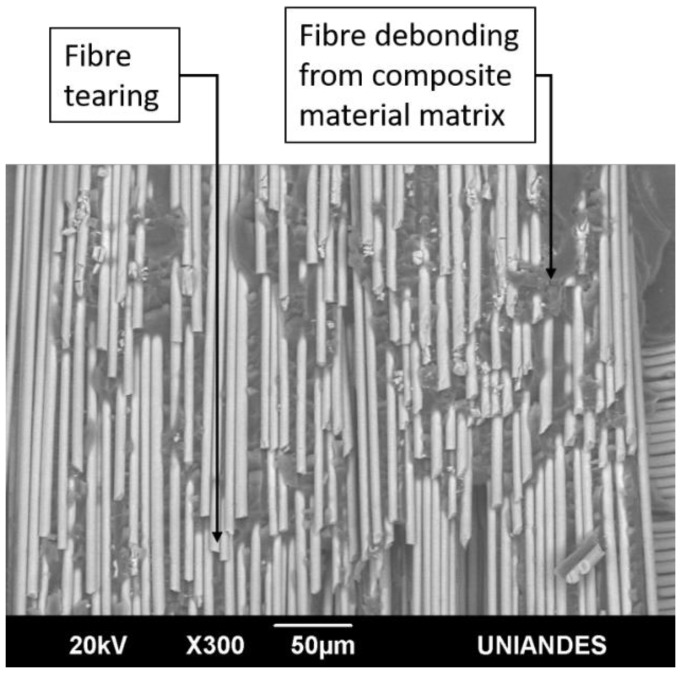
SEM fracture surface image of the 6.35-mm-thick I3 DCB specimen tested under impact fatigue.

Figure 18 and Figure 19 show the SEM images of the fracture surface of two DCB specimen tested under constant amplitude fatigue and impact fatigue respectively: tearing and debonding of the fibre from the composite material matrix are observed in both. According to ASTM D5573 [31], this type of failure occurs within the FRP substrate near the surface, which is characterised by a thin layer of the FRP resin matrix on the adhesive, with few glass fibres transferred from the substrate to the adhesive; it is classified as fibre-tear failure. This result indicates that an adhesive bond is adequate due to the capability of the adhesive to delaminate the substrate.

## 4. Discussion

Diagrams of the mode I crack propagation rate da/dN as a function of the maximum strain energy release rate were obtained for DCB specimens under repeated impact and constant amplitude fatigue. The modified Paris law equation for all of the examined thicknesses of the adhesive layers reveals no significant difference among adhesive thicknesses under both types of dynamic load induced on the specimens, i.e., repeated impact and constant amplitude fatigue, respectively. This result is caused by the fact that the propagation of the crack between the substrate plies occurs in a noncohesive manner; hence, the mode I critical strain energy release rates are similar for the examined thicknesses of the three adhesive layers. The same failure mechanism is observed during the determination of the critical strain energy release rate; accordingly, significant effects of the adhesive bond line thickness neither on the crack propagation rate nor on the critical strain energy release rate of the joint are not observed. This can be attributed to the plastic zone formed at the crack tip, which is completely developed under the intervals of the adhesive thicknesses tested and not enough restrictions of the adherends are done regarding the plastic deformation at the crack tip. Total development of the plastic zone is between 0.25–0.5 mm, which are values much lower than those use in the aircraft [32,33]. Notably, no significant relation between the adhesive bond line thickness and the previously explained crack propagation parameters is only valid for this specific joint configuration, considering the material type, manufacturing process, stacking sequence and substrate geometry.

**Figure 20:**
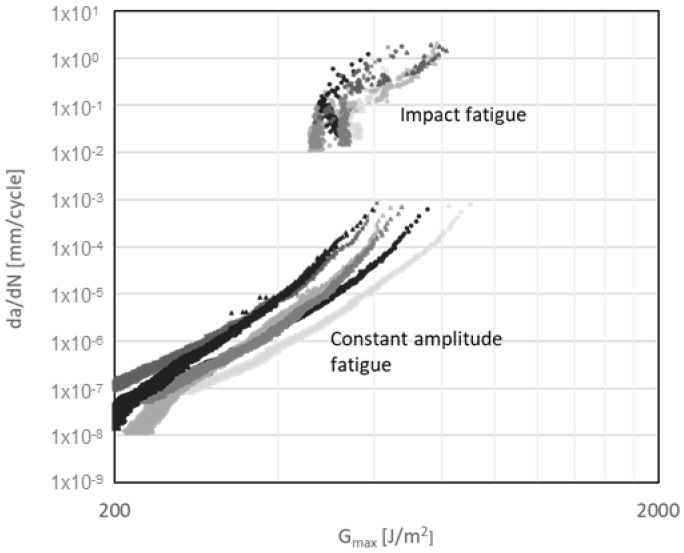
G_Imax_ vs. da/dN diagram for DCB specimens subjected to impact and constant amplitude fatigue.

A fatigue sensitivity expression was proposed in [34], corresponding to the ratio of the maximum strain energy release rate threshold G_th_ to the critical strain energy release rate G_IC_, i.e., G_th_/G_IC_. As the fatigue sensitivity is lower, a crack is more susceptible to propagate under low strain energy release rates; thus, it is a parameter that provides an overview of the susceptibility of the crack to grow under load conditions. The fatigue sensitivity values obtained for specimens tested under repeated impact are 0.46, 0.37 and 0.35 for adhesive thicknesses of 3.18 mm, 6.35 mm and 12.70 mm, respectively; thus, cracks are more susceptible to grow with thick adhesives under low-energy, repeated impact. On the other hand, the fatigue sensitivity values obtained for specimens tested under constant amplitude fatigue are 0.13, 0.17 and 0.16 for adhesive thicknesses of 3.18 mm, 6.35 mm and 12.70 mm, respectively, indicating that significant differences in terms of fatigue sensitivity among different adhesive thicknesses for those specimens tested under constant amplitude fatigue are not observed. Moreover, from fatigue sensitivity results, the crack is more susceptible to propagation under constant amplitude fatigue loading conditions; nevertheless, at the same interval of the maximum strain energy release rate da/dN, the crack growth velocity is three orders of magnitude higher for specimens tested under repeated impact (Figure 20). High load rates in polymeric-based adhesives lead to an increase in their mechanical strength [35]; hence, it could be related to the diameter of the plastic zone at the crack tip. Large plastic deformation at the crack tip generates a large damage zone; thus, most of the energy applied under dynamic regimes is absorbed in plastic deformation. Under the impact fatigue regime, cracks propagate under high load rates, with less development of the plastic zone; therefore, the material exhibits a more brittle behaviour, accounting for the accelerated crack growth under repeated impact [36]. The comparison of the constant m of the modified Paris law, which is only valid for region II, between constant amplitude and impact fatigue testing reveals no significant variation; in addition, this result could be related to the fact that low loading rates decrease the maximum strain energy release threshold due to the increment of the damage area as explained previously.

The impact fatigue crack propagation rate was measured previously [37] using lap strap joint specimens of CRFP substrates and an EA 9628 adhesive, and a fatigue sensitivity of 0.3 was reported; this value is similar to that obtained herein. In addition, similar results were reported for the crack propagation rate under constant amplitude fatigue. Previously [38], DCB specimens comprising unidirectional composite adherends bonded together with a structural adhesive were examined under a constant amplitude fatigue regime, and a fatigue sensitivity of ~0.1 was obtained. In another study [39], a mode I delamination test also was performed on a carbon-epoxy laminate under constant amplitude fatigue, and a curve of the crack growth rate vs. the normalized strain energy release rate was plotted; a fatigue sensitivity of 0.2 was obtained. In addition, the strain energy release rate at the Paris limit was ~65% of the fracture toughness. The Paris limit for the strain energy release rate in this study was ~70% of the critical strain energy release rate; this value is in agreement with that reported previously [37]. This change in the slope of the log–log diagram (da/dN vs. G_Imax)_ before G_IC_ could be related to dynamic parameters, such as strain rate, load frequency and stress ratio, that affect the behaviour of substrates, which are viscoelastic materials that are sensitive to load rates; moreover, its thickness and configuration make them low-stiffness materials in comparison to steel, which could affect the Paris law limits [40].

## 5. Conclusions

In this study, effects of the adhesive joint thickness of DCB specimens were analysed under quasistatic, cyclic impact and constant amplitude fatigue loading. Crack growth in DCB specimens under mode I quasistatic and dynamic loading regimes followed the same path, i.e., cracks propagated in a cohesive manner in the precrack zone and continued growing between the substrate plies until failure due to the inclusion of very thick adhesive layers and the fact that fracture toughness of the FRP interfaces is lower than the corresponding for the bulk adhesive. Additionally, cracks are more susceptible to propagation under constant amplitude fatigue loading conditions according to the fatigue sensitivity expression indicator; nevertheless, at the same interval of the maximum strain energy release rate da/dN, the crack growth velocity is three orders of magnitude higher for specimens tested under repeated low energy impacts, thus, it is imperative to consider in the fatigue life design of structures this type of loads when they may be subjected to them.

## Data Availability

The data presented in this study are available on request from the corresponding author.
